# Development, Validation, and Application of the UPLC-DAD Methodology for the Evaluation of the Qualitative and Quantitative Composition of Phenolic Compounds in the Fruit of American Cranberry (*Vaccinium macrocarpon* Aiton)

**DOI:** 10.3390/molecules27020467

**Published:** 2022-01-12

**Authors:** Rima Urbstaite, Lina Raudone, Mindaugas Liaudanskas, Valdimaras Janulis

**Affiliations:** 1 Department of Pharmacognosy, Faculty of Pharmacy, Lithuanian University of Health Sciences, 50162 Kaunas, Lithuania; Lina.Raudone@lsmuni.lt (L.R.); Mindaugas.Liaudanskas@lsmuni.lt (M.L.); Valdimaras.Janulis@lsmuni.lt (V.J.); 2 Institute of Pharmaceutical Technologies, Faculty of Pharmacy, Lithuanian University of Health Sciences, 50162 Kaunas, Lithuania

**Keywords:** cranberry, phenolic compounds, validation, *Vaccinium macrocarpon*

## Abstract

Phenolic compounds in the fruit of American cranberry (*Vaccinium macrocarpon* Aiton) determine the antioxidant, anti-inflammatory, anticancer, and other biological effects. The berries are used in the production of medicinal preparations and food supplements, which highlights the importance of qualitative and quantitative analysis of phenolic compounds in cranberry fruit raw material. The aim of our study was to develop and validate an efficient, cost-effective, reproducible, and fast UPLC-DAD methodology for the evaluation of the qualitative and quantitative composition of phenolic compounds in raw material and preparations of American cranberry fruit. During the development of the methodology, chlorogenic acid and the following flavonols were identified in cranberry fruit samples: myricetin-3-galactoside, quercetin-3-galactoside, quercetin-3-glucoside, quercetin-3-α-L-arabinopyranoside, quercetin-3-α-L-arabinofuranoside, quercetin-3-rhamnoside, myricetin, and quercetin. The developed and optimized UPLC-DAD methodology was validated according to the guidelines of the International Council for Harmonization (ICH), evaluating the following parameters: range, specificity, linearity (R^2^ > 0.999), precision (%RSD < 2%), LOD (0.38–1.01 µg/mL), LOQ (0.54–3.06 µg/mL), and recovery (80–110%). The developed methodology was applied to evaluate the qualitative and quantitative composition of phenolic compounds in fruit samples of cranberry cultivars ‘Baifay’, ‘Bergman’, ‘Prolific’, and ‘Searles’, as well as ‘Bain-MC’ and ‘BL-12′ clones. In the tested samples, the majority (about 70%) of the identified flavonols were quercetin derivatives. The greatest amount of quercetin-3-galactoside (1035.35 ± 4.26 µg/g DW) was found in fruit samples of the ‘Searles’ cultivar, and the greatest amount of myricetin-3-galactoside (940.06 ± 24.91 µg/g DW) was detected in fruit samples of the ‘Woolman’ cultivar.

## 1. Introduction

American cranberries (*Vaccinium macrocarpon* Aiton) are perennial evergreen plants of the *Ericaceae* A.L. de Jussie family native to North America that are now widely cultivated in Europe as well [1]. Due to the effect of biologically active compounds of cranberry fruit and their use in the food industry, large cranberry plantations are grown in Lithuanian farms and private gardens [2,3]. The following groups of biologically active compounds have been identified in cranberry fruit: flavonols (quercetin and myricetin derivatives) [4], flavan-3-ols, anthocyanins [5], tannins, derivatives of phenolic acids, and triterpenoids [6].

The biologically active compounds found in cranberry fruit reduce cellular oxidative stress while increasing resistance to H_2_O_2_-induced DNA damage in tissue cells [7]. Cranberry fruit preparations have antibacterial [8], antiviral [9], and anticancer [10,11] effects. The type A trimeric proanthocyanidin complex and flavonols (quercetin and myricetin) in cranberries inhibit the adhesion of the uropathogenic strain of *Escherichia coli*, the causative agent of urinary tract infections, to the receptors of the urinary tract epithelial cells [12], and thus cranberry fruits and their preparations are used for the prevention and treatment of urinary tract infections [13]. Chlorogenic acid is prevalent in botanical raw materials, including cranberry fruit. Studies have shown that chlorogenic acid has antihypertensive, antidiabetic, antiobesity, and antidyslipidemic effects [14]. Quercetin determined in cranberry fruit has an effect on bladder [15], breast [16], and ovarian [17] cancer cells. The anticancer effects of quercetin have been associated with its ability to selectively inhibit cell proliferation and induce cancer cell death without harming healthy cells [18]. Quercetin has been shown to inhibit influenza viral infection by stimulating the cellular immune system and inhibiting viral mechanisms of action [19].

Quality assurance of botanical raw materials and preparations is important for the production and effective use of cranberry fruit preparations and food supplements in medical practice. In order to achieve that objective, it is expedient to determine the composition of the active ingredients so that the consumers receive quality products. Such demand increases with the increasing use of the botanical raw material of cranberries and the development and production of food products, food supplements, functional foods, and medicinal products [7,20]. The qualitative and quantitative composition of biologically active compounds of the botanical raw material of cranberries and their preparations is determined by climatic conditions, the time of the preparation of the raw material, the storage conditions of the botanical raw material, and the peculiarities of its processing. According to literature, about 200 cultivars of American cranberries are grown, with varying fruit yield and the composition of biologically active compounds [21]. Assurance of the quality control of medicinal plant raw materials and preparations requires the development of modern methodologies for the phytochemical analysis of such materials that would be in line with the scientific progress and would allow the evaluation of the qualitative and quantitative content of biologically active compounds at the lowest costs of labor and time.

Determination of the phytochemical profile and identification of the markers are important for quality assurance and control of cultivated or naturally growing cranberry fruit. In the matrix of biologically active compounds of cranberries, a specific chromatographic profile of flavonol glycosides may be isolated, the identification of which could be used to assess the quality of cranberry raw material and preparations [22]. It would be expedient to develop a methodology for qualitative and quantitative analysis to test the authenticity of the botanical raw material of morphologically similar plants of the *Ericaceae* family (*Vaccinium macrocarpon* Aiton., *Vaccinium oxycoccus* L., *Vaccinium vitis-idaea* L., and *Arctostaphylos uva-ursi* L.). Plants of the cranberry species *Vaccinium macrocarpon* and *Vaccinium oxycoccus* are genetically and morphologically similar, and thus the chromatographic profile of the biologically active compounds in their fruit samples is similar, yet the quantitative composition of phenolic compounds differs [23]. The evaluation of the variation in the qualitative and quantitative composition of phenolic compounds may explain the peculiarities of the therapeutic effect of the preparations [24], while at the same time allowing for determining the identity and quality of the raw material [25].

Different methods of instrumental analysis have been mentioned in publications on the evaluation of phenolic compounds in the botanical matrix. The methods for the evaluation of the total amount of phenolic compounds in samples of botanical raw materials that have been described in scientific literature are created and developed on the basis of the spectrophotometric method and cannot be used to evaluate the qualitative and quantitative content of individual phenolic compounds [26].

Different methods based on chromatographic methods can be used for the qualitative and quantitative analysis of phenolic compounds in botanical raw materials. Cre et al. [27] have developed an ultra-high-performance thin-layer chromatography technique for the evaluation of hyperoside and chlorogenic acid in cranberry fruit samples. The advantages of thin-layer chromatography are a simple preparation of the samples, short analysis time, and the ability to analyze multiple samples simultaneously. However, no other flavonoids were identified in cranberry raw material [27]. Thin-layer chromatography is intended for qualitative analysis and is therefore not suitable for the qualitative and quantitative evaluation of the entire chromatographic profile of flavonoids [28,29].

HPLC (high-performance liquid chromatography) method is an instrument of choice for the evaluation of the distribution of secondary metabolites of the plant matrix. The methodologies developed on the basis of this method are applied for qualitative and quantitative evaluation of the profile of flavonoids in botanical raw materials [30,31]. The advantages of the HPLC method are that it only requires a small amount of the test sample, the efficient distribution of the plant matrix components, and a reproducible and fast process of qualitative and quantitative analysis. The disadvantages of the method are the following: the analysis requires expensive equipment serviced by qualified staff; the preparation of the test sample for analysis consists of several procedures; the selection of the gradient or isocratic elution systems and conditions, and larger amounts of organic solvents used for the separation of the analytes [32]. Liquid chromatography with MS detection is used for the evaluation of the flavonoid structure [33]. Quantitative and qualitative evaluation of flavonoids is performed by applying the liquid chromatography method with a DAD detector based on the ability of the test compound to selectively absorb UV rays in proportion to the concentration in the sample [34]. Flavonols have a UV absorption spectrum, thus the use of a DAD detector is more appropriate for routine testing and reliable analytical results than MS/MS detection [28].

To ensure safer and more ecological working conditions when conducting phytochemical studies of medicinal plant raw materials, there is a continuous search for more ecological research techniques, and new analytical methods that meet these requirements are being developed and validated. In order to limit the use of organic solvents, it is expedient to develop methodologies that use less harmful solutions [35]. UPLC (ultra-high-performance liquid chromatography), compared to HPLC, is a better alternative because it is less harmful to the environment due to the lower amount of organic solvents used, has a shorter analysis time, and is characterized by higher sensitivity and resolution [36,37]. The methods developed and validated on the basis of UPLC can be used for the qualitative and quantitative analysis of biological matrices, and therefore our study aimed to develop a short, simple, accurate, and efficient methodology suitable for routine qualitative and quantitative studies of medicinal plant raw material samples which would employ lower amounts of harmful organic solvents, thus reducing environmental pollution.

The developed and validated UPLC-DAD methodology can be applied to routine studies on the identification of cranberry raw material and the identification and quantification of myricetin and the quercetin group compounds in it. Our proposed research methodology was used for a qualitative and quantitative analysis of the fruit of American cranberry cultivars ‘Baifay’, ‘Bergman’, ‘Prolific’, ‘Searles’, and ‘Woolman’, as well as genetic clones ‘Bain-MC’ and ‘BL-12’. The study helped to determine analyte profiles in chromatograms of the predominant biologically active compounds (chlorogenic acid and identified flavonols) in cranberry fruit.

## 2. Results and Discussion

### 2.1. Development of the Method 

The development and validation of the method are interrelated processes that demonstrate the scientific reliability of the qualitative and quantitative evaluation of analytes in the developed methodology [38]. The aim of this study was to develop a UPLC-DAD methodology for the reliable, rapid, and accurate evaluation of phenolic compounds in the ethanol extract of cranberry fruit.

Reverse-phase columns are commonly used for the separation of phenolic compounds due to the polarity of the molecules [39,40]. During the development of the method, analyte separation was performed using an ACQUITY UPLC BEH C18 (2.1 × 50 mm, 1.7 µm) and ACE C18 (100 × 2.1 mm, 1.7 µm) reversed-phase columns. The ACE C18 reversed-phase column (100 × 2.1 mm, 1.7 µm) was chosen for further analysis due to a better separation of the analytes and their greater symmetry. Even though the properties of methanol and acetonitrile are similar, the water and acetonitrile gradient was chosen as the mobile phase [41] because compared to the mobile phase composed of the mixture of methanol and water, the use of a mixture of water and acetonitrile as the mobile phase reduces the elution time, enhances the symmetry of the last peaks of analytes eluted from the column, and allows for a more accurate quantification of the compounds at low concentrations [5]. Flavonoids can be ionized, which reduces their adsorption to the stationary phase of the column, and peak tailing of analytes can occur in the chromatogram [42]. In order to reduce the ionization of the matrix components and to improve the resolution and recovery of the analysis, the aqueous phase was acidified with formic acid [5].

The selection of the appropriate column temperature is one of the parameters of liquid chromatography that affects the retention time of the analytes and the efficiency of the separation. Flavonoid separation is usually performed at a column temperature of 25–35 °C, and such temperature fluctuations do not significantly affect the separation of the analytes [43]. As the temperature increases, the viscosity of the solvents decreases, resulting in a faster separation of the analytes in the column, and thus sometimes, to reduce the duration of the analysis, the column temperature is maintained at >40 °C, yet often this reduces the efficiency of the distribution of phenolic compounds [36,44]. Based on the literature data, a column temperature of 30 °C was used for the analysis [43,45].

During the study, by varying the mobile phase gradient consisting of 0.1–1% formic acid (mobile phase A) and 100% acetonitrile (mobile phase B), the flow rate (0.4–0.5 mL/min), and the duration of the analysis (from 10 to 15 min), we aimed at optimizing the isolation of the analytes of the compounds in the cranberry fruit extract at the wavelength of 360 nm. During the study, the injection volume ranged from 1 to 10 µL. The injection volume of 1 µL was chosen for further analysis because with larger injection volumes, the adjacent peaks were merged. During the evaluation of the analytes of phenolic compounds in the chromatogram of cranberry fruit extracts, the best sensitivity and resolution were observed when using a gradient composed of 0.1% formic acid (A) and 100% acetonitrile (B) at a flow rate of 0.5 mL/min and the following gradient change: 0 min, 95% A; 1 min, 88% A; 3 min, 88% A; 4 min, 87% A; 9 min, 75% A; 10.5 min, 70% A; 12 min, 70% A; 12.5 min, 10% A; 13 min, 10% A; 13.5 min, 95% A; 14.5 min, 95% A, delaying the next injection for 2 min to stabilize the initial 95% A and 5% B concentrations. Using this gradient, after an injection of 1 µL of cranberry fruit extract, phenolic acid analytes were isolated within 0 to 5 min, and the analytes of flavonol compounds were isolated within 6 to 13 min.

### 2.2. Specificity

**Figure 1:**
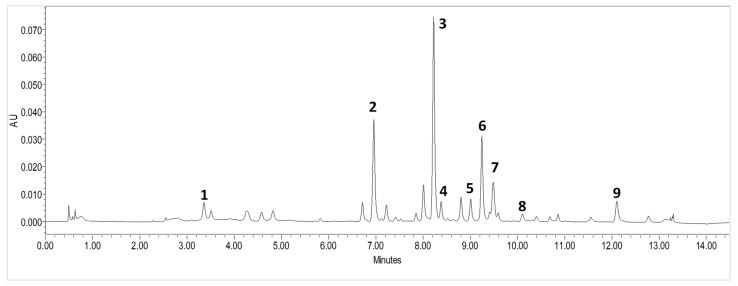
UPLC-DAD profile of American cranberry fruit samples at 360 nm. (1) chlorogenic acid, (2) myricetin-3-galactoside, (3) quercetin-3-galactoside, (4) quercetin-3-glucoside, (5) quercetin-3-α-L-arabinopyranoside, (6) quercetin-3-α-L-arabinofuranoside, (7) quercetin-3-rhamnoside, (8) myricetin, and (9) quercetin.

The identification and qualitative analysis of the compounds were performed by comparing the UV absorption spectrum of the reference standard with the UV absorption spectrum of the matrix peaks of American cranberry, using the same retention time [46]. During the study, the following compounds were identified: (1) chlorogenic acid and eight flavonols, (2) myricetin-3-galactoside, (3) quercetin-3-galactoside, (4) quercetin-3-glucoside, (5) quercetin-3-α-L-arabinopyranoside, (6) quercetin-3-α-L-arabinofuranoside, (7) quercetin-3-rhamnoside, (8) myricetin, and (9) quercetin (Figure 1). The retention times of the compounds and the values of the peaks in the UV-vis absorption spectrum are given in Table 1. The determined profile of the cranberry raw material is specific to cranberries, which was confirmed by the data provided in previous scientific publications, in which a similar flavonol profile was obtained when applying the HPLC analysis method that lasted up to 3.5 times longer [4,31].

Among the compounds identified, quercetin derivatives predominated, accounting for the majority of flavonols in the studied cranberry samples [30,31]. Quercetin and myricetin derivatives have similar absorption spectra with two maxima, and thus reference standards are necessary for the identification of the compounds [31]. In our study, we did not detect compounds identified by other investigators, such as myricetin-3-rhamnoside [30], quercetin-3-rutinoside [2,47,48], and kaempferol-3-glucoside [49], possibly due to a too-low concentration of these compounds in the tested matrix. Synapic acid (Rt 7.591 min) and trans-ferulic acid (Rt 7.374 min) were identified in the study, and their amounts in the studied American cranberry matrix were below the LOQ value.

### 2.3. Linearity and LOD and LOQ

The linear range of the identified compounds, calibration equations, their coefficients of determination, and the range of qualitative and quantitative evaluation are presented in Table 2. The linear range of detection of the identified compounds was from 0.78 to 200 µg/mL and included all concentrations of the phenolic compounds identified during the study. The coefficients of determination (R^2^) for all analyte calibration curves were greater than 0.999, confirming the linearity of the optimized UPLC methodology [38,50]. The calculated limits of detection (LOD) of the target analytes ranged from 0.38 to 1.01 µg/mL, and the limit of detection (LOQ) ranged from 0.54 to 3.06 µg/mL.

### 2.4. Accuracy

The accuracy of the method of the analysis is expressed in percentage and indicates the proximity between the measured value and the actual value of the analyte [51,52]. Accuracy should be assessed using a certified reference plant material or a matrix without the target analyte [53,54]. In the absence of certified reference cranberry fruit raw material, accuracy was assessed by applying two alternative methods [53]. The first method estimates the marginal recovery based on the amount of the reference standard added to the blank matrix, while the second method estimates the total recovery based on the addition of the exact amount of the reference standard into the matrix of a natural analyte of a known concentration (Table 3) [53]. Marginal recovery was assessed within the linearity range at three concentration levels (low, medium, or high). The recovery determined at level 1 was 97.05–105.40%; at level 2, it was 96.17–103.59%, and at level 3, it was 95.97–103.59%, the relative standard deviation ranging from 0.03 to 3.16%.

Total recovery was determined by adding the exact amount of the analytical standard to the natural matrix at a 2:1 ratio to increase the concentration of the target analyte in the matrix by 50, 100, or 150% [54]. The recovery at level 50% was 94.10–107.40%; at level 100%, it was 96.33–108.13%, and at level 150%, it was 93.64–106.84%, with RSD% ranging from 0.02 to 4.37%. 

The accuracy assessed by both methods did not exceed the limit of 80–110% specified in the European Commission Directive 96/23/EC [55]. The comparison of the results of both methods showed that the results obtained in the case of the marginal recovery were within the narrower range of 95.97–105.40%, compared to the total recovery range of 93.64–108.13%. Larger deviations in total recovery may have been influenced by the matrix effect, which changed the intensity of the predicted response when the analyte interacted with other components in the matrix [56,57].

### 2.5. Precision

The precision of the UPLC-DAD methodology was evaluated according to two parameters: repeatability and intermediate precision (or intra-day precision and inter-day precision). The obtained parameter values are presented in Table 4. Relative standard deviations for retention time ranged from 0.10 to 0.20% for intra-day precision and from 0.07 to 0.17% for inter-day precision. The RSD% for repeatability of the identified phenolic compounds ranged from 0.52 to 1.48%, and the RSD% for intermediate precision ranged from 0.59 to 1.88%. The RSD% of the intermediate precision did not exceed the value range of the RSDr% calculated according to the Horwitz equation. The content of phenolic compounds in the matrix of American cranberry fruit makes up a small part of all the components of the matrix, and therefore the calculated permissible values of RSDr% deviation were higher (5.67–8.09%) than the 2% deviation limit often recommended in literature [50,53,58,59].

### 2.6. Determination of Phenolic Compounds in Fruit Samples of American Cranberry Cultivars 

**Figure 2:**
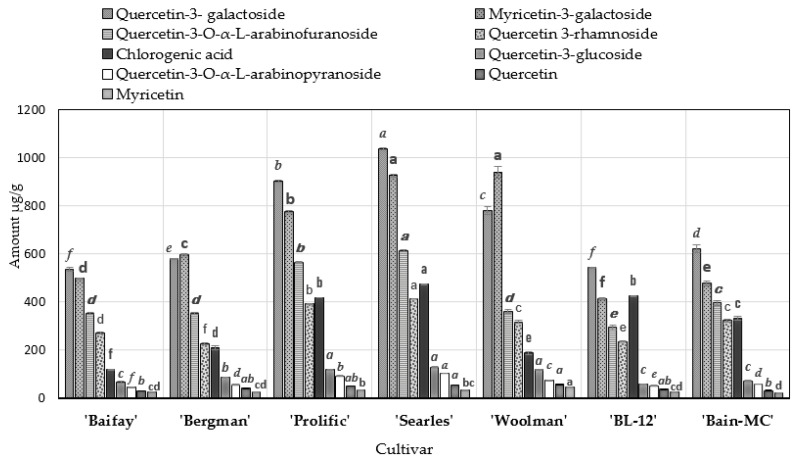
Variation in the amounts of phenolic compounds in fruit samples of American cranberry cultivars. Letters of different fonts and sizes indicate statistically significant differences between fruit samples of different cranberry cultivars (*p* < 0.05).

A validated methodology for the determination of the quantitative and qualitative composition of phenolic compounds based on the UPLC-DAD method was used for the evaluation of the composition of biologically active compounds in fruit samples of American cranberry cultivars ‘Baifay’, ‘Bergman’, ‘Prolific’, ‘Searles’, and ‘Woolman’ as well as in genetic clones ‘Bain-MC’ and ‘BL-12′. The results of the quantitative composition of the detected phenolic compounds in the studied fruit are presented in Figure 2. The results of the study suggest that representatives of the flavonol group (quercetin-3-galactoside and myricetin-3-galactoside) predominated in the studied fruit samples.

Samples of the ‘Searles’ cultivar had the highest content of quercetin-3-galactoside (1035.35 ± 4.26 µg/g DW) (*p* < 0.05). Maximum levels of myricetin-3-galactoside (*p* < 0.05) were found in fruit samples of cranberry cultivars group of ‘Searles’ and ‘Woolman’ (mean 932.64 ± 15.27 µg/g DW). Meanwhile, the lowest content of quercetin-3-galactoside (535.43 ± 7.43 µg/g DW) was found in fruit samples of cranberry cultivar ‘Baifay’ (*p* < 0.05). The lowest (414.24 ± 2.32 µg/g DW) content of myricetin-3-galactoside was found in the fruit samples of the American cranberry genetic clone ‘BL-12’ (*p* < 0.05).

The fruit samples of the American cranberry cultivar ‘Searles’ was found to have the highest levels of quercetin-3-α-L-arabinofuranoside (613.80 ± 3.66 µg/g DW), quercetin-3-rhamnoside (413.68 ± 1.13 µg/g DW), and quercetin-3-α-L-arabinopyranoside (104.63 ± 0.98 µg/g DW) (*p* < 0.05). The highest levels of quercetin-3-glucoside were detected in fruit samples of cranberry cultivars group of ‘Prolific’, ‘Searles’, and ‘Woolman’ (means 122.46 ± 1.46 µg/g DW) (*p* < 0.05). The lowest (293.79 ± 8.99 µg/g DW) content of quercetin-3-α-L-arabinofuranoside was found in the fruit samples of the cranberry genetic clone ‘BL-12’ (*p* < 0.05). The lowest (227.49 ± 4.03 µg/g DW) content of quercetin 3-rhamnoside (*p* < 0.05) was found in the fruit samples of the cranberry cultivar ‘Bergman’.

Our methodology showed that in the studied cranberry fruit samples, quercetin accounted for about 1.35–2.00%, and myricetin for 0.87–1.60% of the total amount of the identified flavonols. The highest amounts of quercetin (57.73 ± 1.36 µg/g DW) and myricetin (46.09 ± 0.60 µg/g DW) were found in fruit samples of the cranberry cultivar ‘Woolman’ (*p* < 0.05).

The highest content of chlorogenic acid (472.97 ± 1.44 µg/g DW) was found in fruit samples of the cultivar ‘Searles’, while the minimum content (119.14 ± 1.80 µg/g DW) was detected in fruit samples of the cultivar ‘Baifay’ (*p* < 0.05). Ruse et al. [60] conducted a study where they found that the amount of chlorogenic acid in the fruit of the ‘Bergman’ cultivar of American cranberry was 260 µg/g. Compared to the results of their study, we detected slightly lower levels of chlorogenic acid (209.61 ± 6.45 µg/g DW) in fruit samples of the ‘Bergman’ cranberry cultivar [60].

Oszmiański et al. [61] used LC/MS QTOF and UPLC-PDA-FL methodologies for qualitative and quantitative analysis of cranberry fruit samples. The authors identified 14 compounds of the flavonol group: 4 myricetin derivatives and 10 quercetin derivatives [61]. The study showed that quercetin-3-galactoside levels in samples of lyophilized fruit of ‘Stevens’, ‘Ben Lear’, and ‘Pilgrim’ cultivars were higher than those of quercetin-3-rhamnoside: accordingly, 771 and 700 µg/g; 1378 and 522 µg, and 806 and 484 µg/g [61]. In their study, Zheng et al. [62] found that the level of quercetin 3-rhamnoside in the fruit of the cranberry cultivar ‘Ben Lear’ was lower (41.6 ± 3.50 µg/g).

Oszmiański et al. [61] found four myricetin derivatives in cranberry fruit samples, the amounts of which were three times higher than those of the quercetin group derivatives. Our study found that about 70% of the compounds identified in cranberry fruit samples were quercetin group glycosides. The results of our study were confirmed by those obtained in a study by Vvedenskaya et al. [63], where quercetin glycosides accounted for up to 80% of flavonols in cranberry fruit samples.

Wang et al. [31] used HPLC MS/MS in their study and detected myricetin-3-galactoside, myricetin-3-arabinofuranoside, quercetin-3-galactoside, quercetin-3-glucoside, quercetin-3-xylopyranoside, quercetin-3-arabinopyranoside, quercetin-3-arabinofuranoside, quercetin-3-rhamnopyranoside, and quercetin. The authors conducted a qualitative and quantitative analysis of the fruit samples of ‘Early Black’, ‘Howes’, ‘Ben Lear’, ‘Stevens’, ‘Crimson Queen’, ‘Demoranville’, and ‘Mullica Queen’ cultivars. In their study, quercetin-3-galactoside was found to account for approximately 31–46% of the total amount of flavonols, myricetin-3-galactoside—for 19–32%, quercetin-3-arabinofuranoside—for 7–17%, and quercetin-3-rhamnopyranoside—for 7–14% of the total amount of flavonols [31]. In the samples of our studied cranberry cultivars, quercetin-3-galactoside accounted for 28.93–32.68%, myricetin-3-galactoside for 23.83–30.37%, quercetin-3-arabinofuranoside for 13.35–19.74%, and quercetin-3-rhamnoside—for 11.55–16.09% of the total flavonol content. 

**Figure 3:**
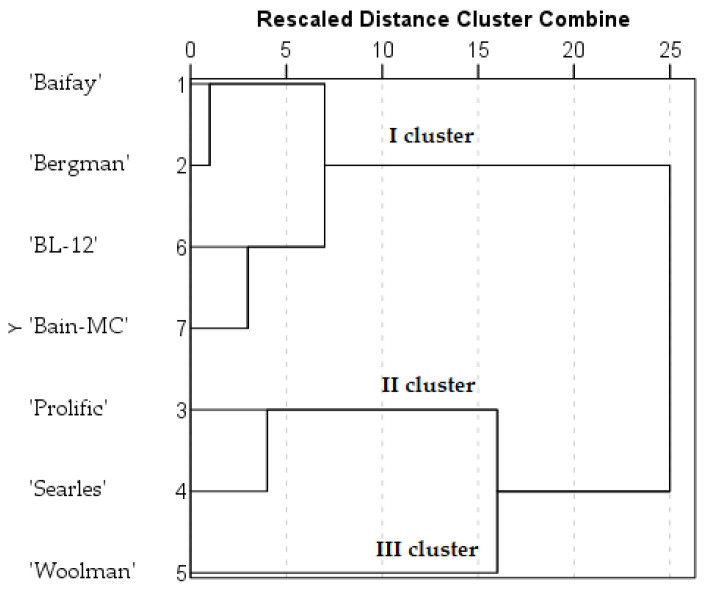
A dendrogram of the distribution of American cranberry cultivars into similar clusters according to the chlorogenic acid and flavonols content in fruit samples.

The analysis of the similarity of the composition of chlorogenic acid and flavonols in American cranberry cultivars was carried out by performing a hierarchical cluster analysis. The cluster analysis of the samples of American cranberry cultivars was performed on the basis of the quantitative composition of identified phenolic compounds. Fruit samples of cranberry cultivars were divided into three clusters (Figure 3). Fruit samples of cranberry cultivars ‘Baifay’, ‘Bergman’, and genetic clones ‘BL-12’ and ‘Bain-MC’ were assigned to cluster I. Fruit samples of cranberry cultivars ‘Proliflic’ and ‘Searles’ were assigned to cluster II. The cultivars of cluster I total amount of quantified phenolic compounds was up to 1.9 times lower than the total amount of quantified phenolic compounds of cultivars of the cluster II. Cluster III consisted of one American cranberry cultivar ‘Woolman’. The total amount of quantified phenolic compounds in cranberry fruit samples of the ‘Woolman’ cultivar was similar to the total amount of quantified phenolic compounds in samples of cultivars of cluster II, but samples of cultivar ‘Woolman’ samples contained higher levels of myrecetin-3-galactosie than quercetin-3-galactoside.

The amount of flavanols tested and quantified in cranberry fruit samples showed the following decreasing trend: the highest levels were found for quercetin-3-galactoside and myricetin-3-galactoside, followed by quercetin-3-α-L-arabinofuranoside > quercetin 3-rhamnoside > quercetin-3-glucoside > quercetin-3-α-L-arabinopyranoside > quercetin > myricetin. Chromatogram profiles of the studied American cranberry cultivars ‘Baifay’, ‘Bergman’, ‘Prolific’, ‘Searles’, and ‘Woolman’, as well as ‘Bain-MC’ and ‘BL-12′ genetic clones, were identical, but varied in the size of the areas of the analyte peaks (Supplementary Figures S6 and Figure S7). Quantitative analysis of flavanols in cranberry fruit samples revealed that the coefficient of variation (CV) of flavanols (quercetin-3-galactoside, myricetin-3-galactoside, quercetin-3-α-L-arabinofuranoside, quercetin-3-rhamnoside, quercetin-3-glucoside, quercetin-3-α-L-arabinopyranoside, quercetin, and myricetin) ranged from 24.96 to 44.14%. 

The fruits of lingonberry (*Vaccinium vitis-idaea* L.) are morphologically similar to those of cranberry. It is especially important to have a tool that can be used to identify and separate ground and dried plant raw materials based on their chemical composition, and to identify the components of these raw materials in food products and food supplements. The myricetin-3-galactoside characteristic of the chromatogram profile of cranberry samples is not detected in the chromatogram profile of lingonberries, and this compound is one of the major markers in cranberry fruit chemotaxonomy [64,65]. Quercetin-3-galactoside and myricetin-3-galactoside are the major components of biologically active compounds in cranberry fruit. Studies of the qualitative and quantitative content of these compounds in cranberry samples are important in evaluating the quality of cranberry fruit raw material, food products, and food supplements. 

The fruits of cranberries that grow in natural habitats or are cultivated are used for food and the preparation of various beverages. It is important to evaluate the quantitative composition of flavonoids in fruit samples, in which the total flavonol content of cranberry fruit is typically in the range of 20–30 mg per 100 g of fresh fruit weight [66]. Studies on the content of flavonols of lyophilized fruit samples of different cranberry cultivars showed that the total amount of the identified flavonols varied from 166 to 331 mg/100 g. Recently, lyophilized cranberry fruit and lyophilized fruit powder have been increasingly commonly used in the production of food products (biscuits, chocolate, yogurt, kissels, or beverages) and as a food supplement. The developed methodology may help to assess the qualitative and quantitative content of biologically active compounds. The results of the research are valuable in assessing the quality of cranberry fruit. The use of cranberry fruit raw material with the determined qualitative and quantitative composition of phenolic compounds may help produce high-quality food products and food supplements.

The developed and validated methodology based on the UPLC-DAD method can be applied for the determination of the content of chlorogenic acid and flavonol group compounds in cranberry fruit samples. This short-term, efficient, and selective methodology allows for routine qualitative and quantitative studies of cranberry fruit raw material. Such studies are important in assessing the chemical composition of fruits of the existing and the newly bred cultivars and genetic clones. The qualitative and quantitative analysis of the samples of cranberry fruit raw material provide new knowledge about the phytochemical composition of the fruit of different cranberry cultivars and the possibility of using quality raw material in healthy food, as well as for the development and production of cranberry-containing food supplements.

## 3. Materials and Methods

### 3.1. Reagents

Analytical and chromatographic grade chemicals and solvents were used for this study: ethanol 96% (*v*/*v*) (manufacturer AB Stumbras, Kaunas, Lithuania), acetonitrile (manufacturer Sigma-Aldrich, Steinheim, Germany), methanol (manufacturer Sigma-Aldrich, Steinheim, Germany), and formic acid (manufacturer Merck, Darmstadt, Germany). Reference HPLC standards for chlorogenic acid (3-caffeoylquinic acid), myricetin, quercetin-3-rhamnoside, quercetin-3-α-L-arabinofuranoside, and quercetin-3-α-L-arabinopyranoside were obtained from Sigma-Aldrich (Steinheim, Germany), for quercetin-3-galactoside and quercetin—from Carl Roth (Karlsruhe, Germany), for myricetin-3-galactoside—from Extrasynthese (Genay, France), and the reference standard for quercetin-3-O-glucoside was obtained from Biochemistry (Buchs, Switzerland).

### 3.2. Raw Material

The objects of the study were samples of mature and ripe fruit of different cultivars of American cranberry (Baifay, ‘Bergman’, ‘Prolific’, ‘Searles’, and ‘Woolman’, and genetic clones ‘Bain-MC’ and ‘BL-12’) grown in Lithuanian climatic conditions, in the collection of the Institute of Botany of the Nature Research Center, Mažieji Gulbinai, Vilnius (54°41′36.6″ N 25°21′56.0″ E). The collection time was September 2020. Cranberry fruits were ground and frozen at −60 °C in an ultra-low-temperature freezer (CVF330/86, ClimasLab SL, Barcelona, Spain).

Cranberry fruits were lyophilized at a pressure of 0.01 mbar at a condenser temperature of −85 °C in a Zirbus lyophilizer (Zirbus technology GmbH, Bad Grund, Germany). The lyophilized cranberry fruits were ground into powder using a Retsch GM 200 electric mill (Retsh GmbH, Hahn, Germany). The samples were stored in tightly closed containers in a dark and dry place. Loss on drying was determined using the method described in the European Pharmacopoeia Ph.Eur.01/2008: 20232 [67].

### 3.3. Preparation of Cranberry Extracts

About 1 g (precise weight) of the lyophilized cranberry powder was extracted with 20 mL of 70% (*v*/*v*) ethanol in an Elmasonic P ultrasonic bath (Singen, Germany) for 15 min at 80 kHz and 565 W at 20–22 °C temperature. After the extraction, the ethanolic extracts were filtered into a 25 mL volumetric flask. The prepared extracts were stored in dark glass vials at 5–8 °C. Prior to the chromatographic analysis, the ethanol extracts were filtered through filters with 0.20 μm pore size (CHROMAFIL Xtra PTFE-20/13).

### 3.4. Chromatographic Analysis

The analysis of the qualitative and quantitative composition of phenolic compounds in cranberry fruit was performed using a Waters ACQUITY Ultra High-Performance LC system (Water, Milford, MA, USA) with a photodiode array detector. An ACE C18 reversed-phase column (ACT, Aberdeen, UK; 100 × 2.1 mm, 1.7 µm particle size) was used for the separation of the compounds at 30 °C. The injection volume was 1 µL, and the distribution was performed using 0.1% formic acid (*v*/*v*) (A) and 100% acetonitrile (B) at a flow rate of 0.5 mL/min and the following gradient change: 0 min, 95% A; 1 min, 88% A; 3 min, 88% A; 4 min, 87% A; 9 min, 75% A; 10.5 min, 70% A; 12 min, 70% A; 12.5 min, 10% A; 13 min, 10% A; 13.5 min, 95% A; and 14.5 min, 95% A, delaying the next injection by 2 min. 

### 3.5. Development and Validation of the Method 

The UPLC-PDA method was developed considering the composition of the mobile phase, the elution gradient, the duration of the analysis, the flow rate, the injection volume, the column parameters, and the optimal temperature. For the validation, a methodology was selected in which the flavonol analytes in the American cranberry fruit matrix were best separated from each other in the chromatogram.

The validation of the method was performed according to the guidelines of the International Council for Harmonization (ICH). The acceptance criteria of the validation were selected considering the recommendations of Eurochem, the European Commission Directive 96/23/EC, and Guidelines for Dietary Supplements and Botanicals [51,55,68,69]. The following parameters were evaluated during the validation: accuracy, precision (repeatability and intermediate precision), specificity, limit of detection, limit of quantification, linearity, and range of determination.

Specificity was determined by comparing the analyte retention time and the UV absorption spectrum between the reference standard and the American cranberry matrix (Supplementary Figures S1–S6). Linearity was determined by constructing calibration regression equations consisting of 5 to 7 points and measuring standard solutions of known concentration. To evaluate linearity, the coefficient of determination (R^2^) was set (suitable when R^2^ > 0.999). The limit of detection (LOD) and the limit of quantitation (LOQ) were calculated according to Formulas (1) and (2) (where σ is the residual standard deviation of the regression line, and S is the slope), respectively [51].

LOD = 3.3σ/S (1)

LOQ = 10σ/S (2)

Precision was determined by evaluating the relative standard deviation (RSD%) of the retention time and the amount of 6 American cranberry extracts (Formula (3)). The RSD% of repeatability was calculated from measurements taken on the same day (*n* = 6), and the intermediate precision was determined by analyzing six American cranberry extracts for three consecutive days (*n* = 18). The acceptable precision value was determined by calculating the RSDr% value (Formula (4)) [53,69].

RSD% = SD/m × 100 (3)

RSDr% = 2C^(−0.15)^ (4)

(RSD%—relative standard deviation, SD—standard deviation, m—mean, RSDr%—acceptable precision value, and C—concentration expressed in parts of mass (g/g).

To determine the accuracy of the method, the evaluation of recovery was performed by applying two techniques. The first technique quantified the reference standards at 3 levels: level 1—low concentration, level 2—medium concentration, and level 3—high concentration. Recovery was calculated according to Formula (5) (where X_p_ is the predicted concentration, and X_1_—the measured concentration). Recovery was regarded as adequate when the Recovery% was in the range of 80–110% [55].

Recovery% = X_p_/X_1_ × 100 (5)

The second technique was used to evaluate the recovery of the compounds in the matrix by adding the exact amount of the reference standard to the examined American cranberry extract at a ratio of 2:1. The amount of the standard added to the matrix solution was sufficient to increase the amount of the test compound in the matrix by 0.5-, 1-, or 1.5-fold [55]. The results were evaluated at three levels, based on the percentage increase in the amount of the compound in the matrix (level 1, 50%; level 2, 100%; level 3, 150%) [55,68]. Recovery was calculated according to Formula 6. Recovery was regarded as adequate when the Recovery% was in the range of 80–110% [55].

Recovery% = (X_2_ − X_1_)/X_add_ × 100 (6)

(X_1_—the measured concentration, X_2_—the amount of the test compound in the matrix, and X_add_—the amount of the standard added).

### 3.6. Statistical Analysis

Data analysis and presentations were performed using computer software programs SPSS Statistics 21 (IBM, Armonk, NY, USA) and Microsoft Excel 2016 (Microsoft, Redmond, WA, USA). During the study, arithmetic means, standard deviations (SD), and relative standard deviations (RSD) of the three repeated evaluations were calculated. Linear regression analysis was performed to calculate the coefficient of determination R^2^ and to construct calibration equations for the calculation of the amounts of the identified phenolic compounds. To evaluate the difference in the amounts of phenolic compounds between samples of different American cranberry cultivars, one-way analysis of variance ANOVA with Tukey’s test for multiple comparisons (significance level set at 0.05) was used.

## 4. Conclusions

A short-term efficient UPLC-DAD detection methodology was developed and validated. The proposed methodology is more environmentally friendly due to the lower consumption of eluents compared to the HPLC methods used for the evaluation of qualitative and quantitative composition of phenolic compounds in cranberry fruit samples described in literature. The validation parameters of the methodology (detection limits, specificity, linearity (R^2^ > 0.999), accuracy (%RSD < 2%), LOD (0.38–1.01 µg/mL), LOQ (0.54–3.06 µg/mL), and recovery (93.64–108.13%) met the requirements of the normative documents and confirm the suitability of the methodology for application.

Chromatogram profiles of the studied American cranberry cultivars ‘Baifay’, ‘Bergman’, ‘Prolific’, ‘Searles’, and ‘Woolman’, as well as those of the ‘Bain-MC’ and ‘BL-12′ clones were identical but differed in the area sizes of the analyte peaks. The highest levels of flavonols and chlorogenic acid were found in cranberry fruit samples of the ‘Searles’ cultivar.

## Figures and Tables

**Table 1:** Retention times and UV-vis absorption spectral data of the identified compounds.

Compound	Retention Time	λmax, nm
Chlorogenic acid	3.321	218.5, 243.4, 324.4
Myricetin-3-galactoside	6.917	261.2, 355.0
Quercetin-3-galactoside	8.236	257.6, 355.0
Quercetin-3-glucoside	8.383	255.2, 358.6
Quercetin-3-α-L-arabinopyranoside	8.984	255.2, 353.8
Quercetin-3-α-L-arabinofuranoside	9.193	255.2, 352.6
Quercetin-3-rhamnoside	9.488	255.2, 347.8
Myricetin	10.101	251.7, 371.8
Quercetin	12.104	254.0, 369.4
Chlorogenic acid	3.321	218.5, 243.4, 324.4

**Table 2:** Parameters of linearity, identification, and the limit of detection of the identified phenolic compounds.

Compound	Linear Range (µg/mL)	Calibration Equation	R^2^	LOD (µg/mL)	LOQ (µg/mL)
Chlorogenic acid	1.95–62.5	*y* = 5060*x* + 570	0.9999	0.38	1.16
Myricetin-3-galactoside	0.78–100	*y* = 3450*x* – 396	0.9999	0.18	0.54
Quercetin-3-galactoside	3.13–200	*y* = 4880*x* + 1180	0.9999	1.01	3.06
Quercetin-3-glucoside	3.13–50	*y* = 4160*x* − 61.7	0.9998	0.92	2.78
Quercetin-3-α-L-arabinopyranoside	3.13–50	*y* = 5250*x* + 861	0.9999	0.70	2.12
Quercetin-3-α-L-arabinofuranoside	3.13–50	*y* = 4170*x* – 199	0.9997	0.99	3.03
Quercetin-3-rhamnoside	3.13–50	*y* = 3690*x* + 797	0.9998	0.76	2.29
Myricetin	1.56–50	*y* = 5360*x* – 1240	0.9999	0.45	1.36
Quercetin	3.13–50	*y* = 7450*x* – 1070	0.9999	0.76	2.29

**Table 3:** Determination of the recovery in reference standards and the American cranberry matrix.

Compound	Marginal Recovery			Total Recovery				
	Level (Standard Concentration)	Recovery (%)	RSD (%)	Level	Predicted Amount in the Matrix, µg/mL	Recovered Amount in the Matrix µg/mL (SD)	Recovery (%)	RSD (%)
Chlorogenic acid	Level 1 (5 µL/mL)	97.11	0.99	50%	16.93	17.31 ± 0.07	106.59	1.16
	Level 2 (25 µL/mL)	96.56	0.57	100%	22.58	23.34 ± 0.13	106.71	1.10
	Level 3 (50 µL/mL)	99.00	1.80	150%	28.22	29.37 ± 0.15	106.77	0.86
Myricetin-3-galactoside	Level 1 (5 µL/mL)	105.23	2.64	50%	38.13	38.55 ± 0.39	103.30	2.99
	Level 2 (20 µL/mL)	98.61	1.16	100%	50.84	52.90 ± 0.29	108.13	1.05
	Level 3 (40 µL/mL)	103.59	0.51	150%	63.55	66.15 ± 0.01	106.84	0.02
Quercetin-3-galactoside	Level 1 (5 µL/mL)	103.03	1.81	50%	49.76	50.74 ± 0.10	105.90	0.55
	Level 2 (50 µL/mL)	102.51	0.82	100%	66.35	68.71 ± 0.45	107.11	1.27
	Level 3 (150 µL/mL)	99.94	0.03	150%	82.94	81.15 ± 0.12	96.42	0.25
Quercetin-3-glucoside	Level 1 (5 µL/mL)	101.83	0.79	50%	4.46	4.44 ± 0.06	98.43	4.01
	Level 2 (20 µL/mL)	100.40	0.46	100%	5.95	5.84 ± 0.01	96.33	0.25
	Level 3 (40 µL/mL)	99.44	1.09	150%	7.43	7.29 ± 0.11	96.83	2.61
Quercetin-3-α-L-arabinopyranoside	Level 1 (5 µL/mL)	97.05	0.54	50%	4.94	4.87 ± 0.06	95.99	3.59
	Level 2 (20 µL/mL)	98.67	3.15	100%	6.59	6.58 ± 0.04	99.80	1.34
	Level 3 (40 µL/mL)	95.97	1.30	150%	8.23	7.99 ± 0.02	95.10	0.51
Quercetin-3-α-L-arabinofuranoside	Level 1 (5 µL/mL)	105.40	2.33	50%	23.37	23.42 ± 0.14	100.56	1.77
	Level 2 (20 µL/mL)	103.59	3.16	100%	31.16	32.26 ± 0.02	107.00	0.14
	Level 3 (40 µL/mL)	101.41	2.29	150%	38.96	40.13 ± 0.56	105.03	2.27
Quercetin-3-rhamnoside	Level 1 (5 µL/mL)	103.01	1.96	50%	14.63	14.77 ± 0.04	103.03	0.75
	Level 2 (20 µL/mL)	96.17	0.13	100%	19.50	19.97 ± 0.16	104.83	1.58
	Level 3 (40 µL/mL)	101.06	1.00	150%	24.38	23.45 ± 0.06	93.64	0.45
Myricetin	Level 1 (5 µL/mL)	99.21	0.56	50%	2.18	2.23 ± 0.02	107.40	2.18
	Level 2 (20 µL/mL)	98.67	3.11	100%	2.90	3.01 ± 0.02	107.58	1.54
	Level 3 (40 µL/mL)	98.78	0.78	150%	3.63	3.76 ± 0.07	106.05	2.84
Quercetin	Level 1 (5 µL/mL)	101.19	1.85	50%	4.54	4.45 ± 0.02	94.10	1.07
	Level 2 (20 µL/mL)	100.07	3.25	100%	6.05	6.12 ± 0.05	102.24	1.47
	Level 3 (40 µL/mL)	97.12	1.93	150%	7.57	7.63 ± 0.20	101.42	4.37

**Table 4:** Values of the precision parameters of the UPLC-DAD methodology.

Compound	Mean Amount µg/g DW ± SD	Intra-Day Precision (%RSD, *n* = 6)		Inter-Day Precision (%RSD, *n* = 18)		RSDr%
		Retention Time	Amount	Retention Time	Amount	
Chlorogenic acid	339.41 ± 2.00 ^d^	0.20	0.52	0.17	0.59	6.63
Myricetin-3-galactoside	745.32 ± 8.72 ^b^	0.17	1.43	0.14	1.16	5.89
Quercetin-3-galactoside	961.91 ± 8.47 ^a^	0.13	0.88	0.11	0.88	5.67
Quercetin-3-glucoside	85.82 ± 1.60 ^g^	0.12	1.38	0.11	1.86	8.15
Quercetin-3-α-L-arabinopyranoside	98.70 ± 1.10 ^f^	0.06	0.65	0.10	1.11	7.98
Quercetin-3-α-L-arabinofuranoside	458.51 ± 2.97 ^c^	0.11	0.59	0.10	0.64	6.34
Quercetin-3-rhamnoside	286.56 ± 2.28 ^e^	0.12	0.71	0.09	0.79	6.79
Myricetin	42.98 ± 0.60 ^h^	0.10	1.20	0.09	1.38	9.04
Quercetin	89.76 ± 1.58 ^g^	0.10	1.30	0.07	1.76	8.09

RSD%—relative standard deviation; RSDr%—acceptable value of repeatability for quantity; different letters indicate statistically significant (*p* < 0.05) differences between the studied compounds of cranberry.

## Data Availability

All data generated during this study are included in this article.

## Supplementary Materials

The following supporting information can be downloaded online. Figure S1: UHPLC-PDA chromatogram (λ = 360 nm) of the standard mixture of quercetin-3-galactoside, quercetin-3-α-L-arabinofuranoside, myricetin, and quercetin at different concentrations; Figure S2: UHPLC-PDA chromatogram (λ = 360 nm) of the quercetin-3-galactoside standard at different concentrations; Figure S3: UHPLC-PDA chromatogram (λ = 360 nm) of the myricetin-3-galactoside standard at different concentrations; Figure S4: UHPLC-PDA chromatogram (λ = 360 nm) of the standard mixture of chlorogenic acid and quercetin-3-rhamnoside at different concentrations; Figure S5: UHPLC-PDA chromatogram (λ = 360 nm) of the quercetin-3-α-L-arabinopyranoside standard at different concentrations; Figure S6: UHPLC-PDA chromatogram (λ = 360 nm) of the American cranberry samples extract of cultivar ‘Prolific’; Figure S7: UHPLC-PDA chromatogram (λ = 360 nm) of the American cranberry samples extract of genetic clone ‘Bain-MC’.

## Sample Availability

Not available.
