# Supplementary material for: Development, Validation, and Application of the UPLC-DAD Methodology for the Evaluation of the Qualitative and Quantitative Composition of Phenolic Compounds in the Fruit of American Cranberry (Vaccinium macrocarpon Aiton)
Source: Molecules. 2022 Jan 12;27(2):467. doi: 10.3390/molecules27020467 (PMC8779177; doi:10.3390/molecules27020467)
Supplement: Supplementary file 1 [file molecules-27-00467-s001.zip › molecules-1542005-supplementary.pdf]

Supplementary material:

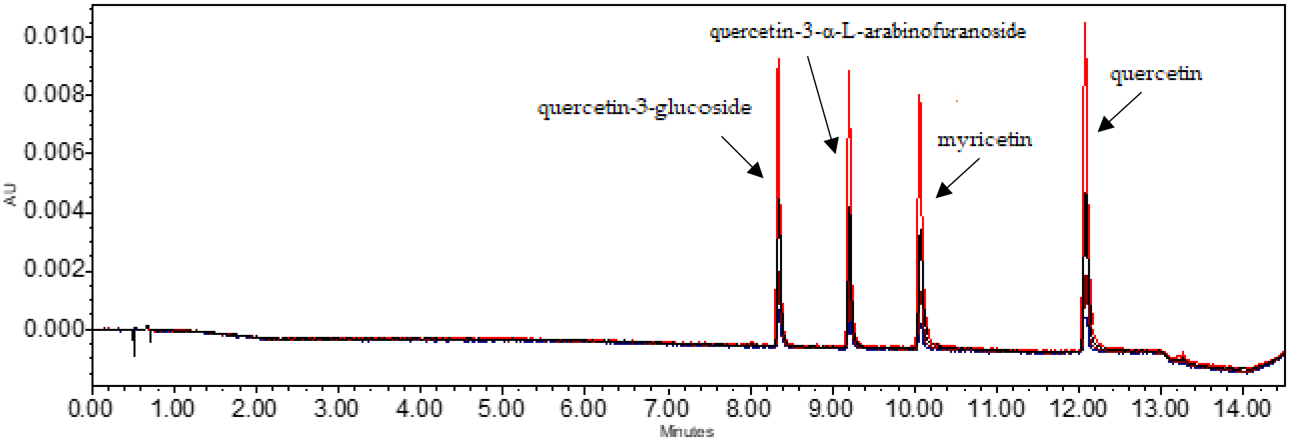

Figure S1: UHPLC-PDA chromatogram (λ = 360 nm) of the standard mixture of quercetin-3-galactoside, quercetin-3-α-L-arabinofuranoside, myricetin, and quercetin at different concentrations.

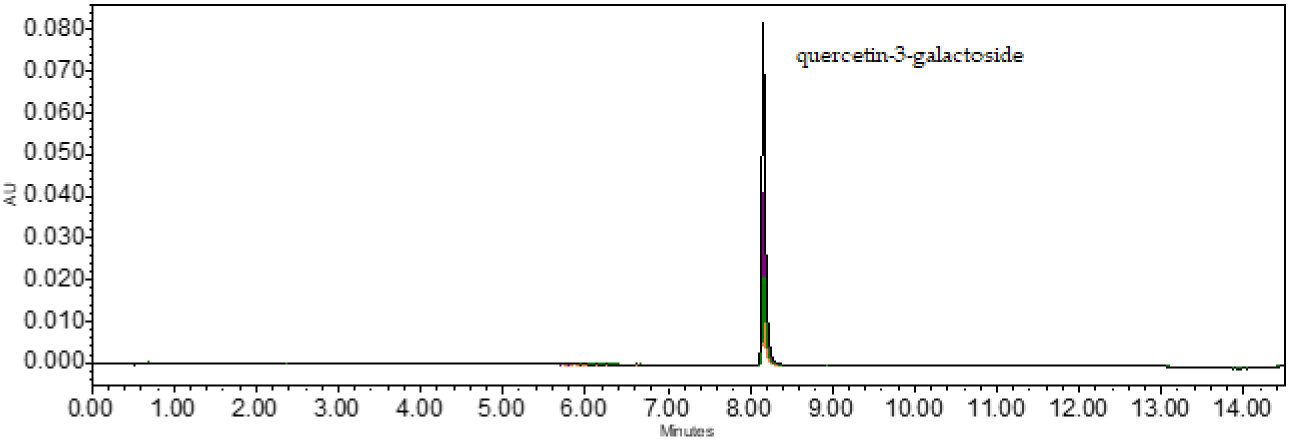

Figure S2: UHPLC-PDA chromatogram (λ = 360 nm) of the quercetin-3-galactoside standard at different concentrations.

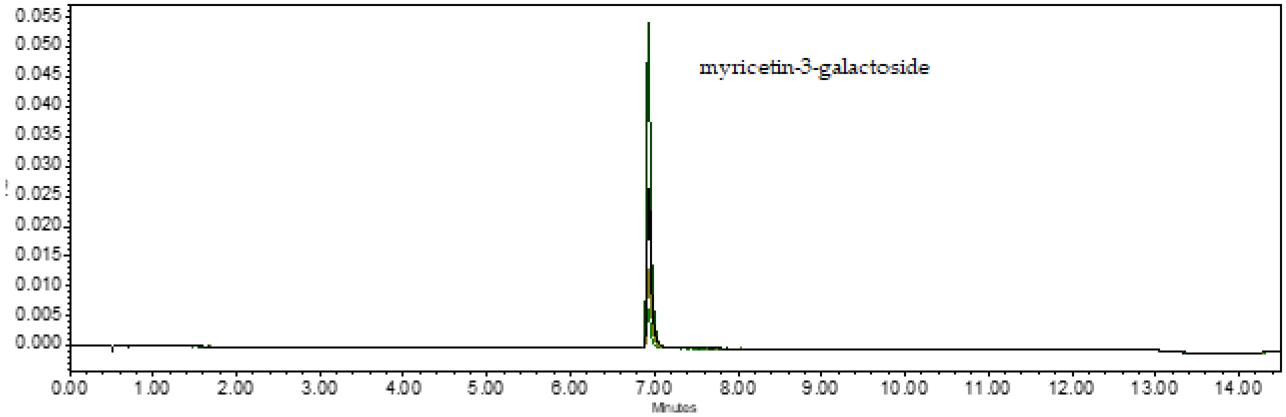

Figure S3: UHPLC-PDA chromatogram (λ = 360 nm) of the myricetin-3-galactoside standard at different concentrations.

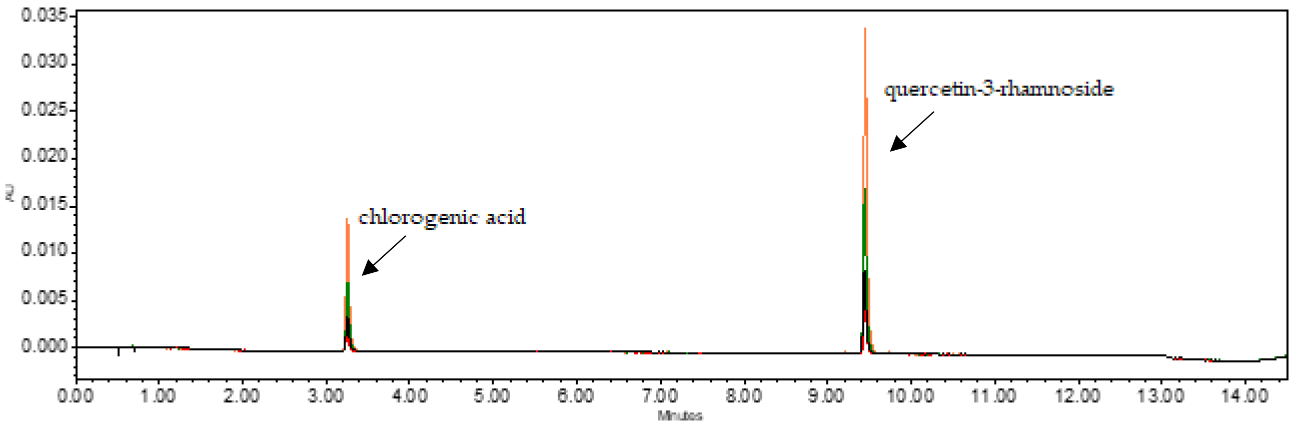

Figure S4: UHPLC-PDA chromatogram (λ = 360 nm) of the standard mixture of chlorogenic acid and quercetin-3-rhamnoside at different concentrations.

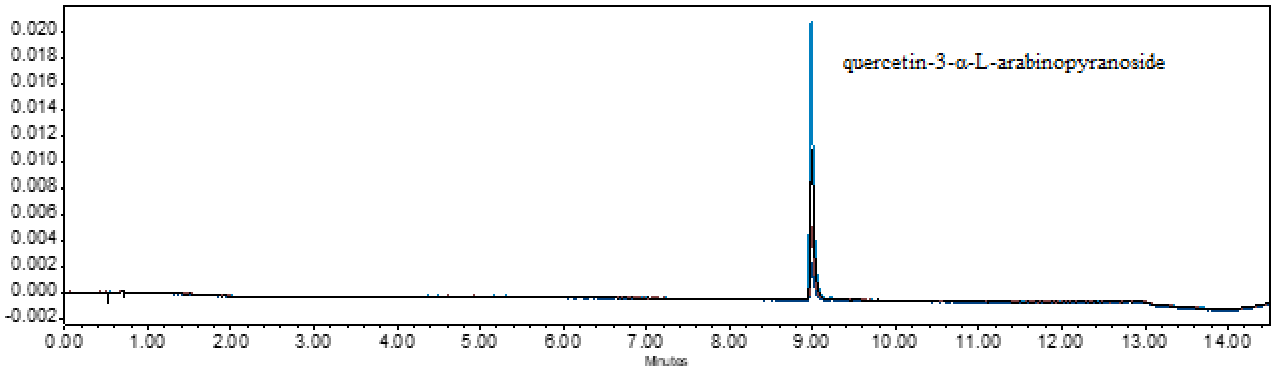

Figure S5: UHPLC-PDA chromatogram (λ = 360 nm) of the quercetin-3-α-L-arabinopyranoside standard at different concentrations.

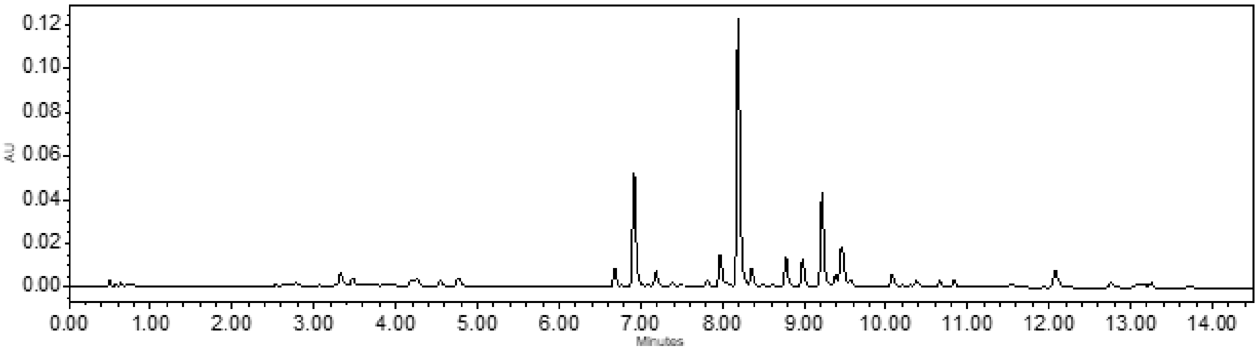

Figure S6: UHPLC-PDA chromatogram (λ = 360 nm) of the American cranberry samples extract of cultivar 'Prolific' .

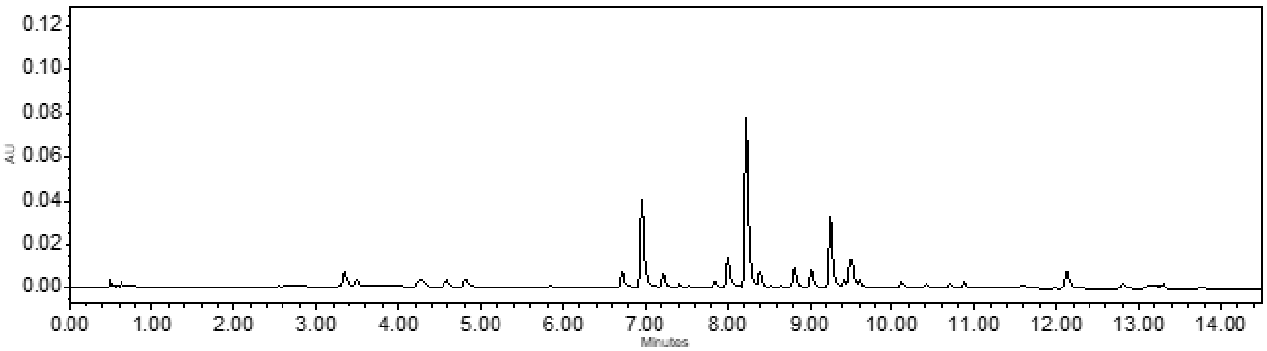

Figure S7: UHPLC-PDA chromatogram (λ = 360 nm) of the American cranberry samples extract of genetic clone 'Bain-MC' .
